# Targeted screening of genetic associations with COVID-19 susceptibility and severity

**DOI:** 10.3389/fgene.2022.1073880

**Published:** 2022-11-30

**Authors:** Ping Li, Yuehua Ke, Wenlong Shen, Shu Shi, Yahao Wang, Kailin Lin, Xinjie Guo, Changjun Wang, Yan Zhang, Zhihu Zhao

**Affiliations:** ^1^ Beijing Institute of Biotechnology, Beijing, China; ^2^ Center for Disease Control and Prevention of PLA, Beijing, China; ^3^ College of Life Science, Henan Normal University, Xinxiang, China

**Keywords:** COVID-19, SARS-CoV-2, targeted capture sequencing, SNP, genetic variant, enhancer, suscepibility

## Abstract

The COVID-19 pandemic has resulted in great morbidity and mortality worldwide and human genetic factors have been implicated in the susceptibility and severity of COVID-19. However, few replicate researches have been performed, and studies on associated genes mainly focused on genic regions while regulatory regions were a lack of in-depth dissection. Here, based on previously reported associated variants and genes, we designed a capture panel covering 1,238 candidate variants and 25 regulatory regions of 19 candidate genes and targeted-sequenced 96 mild and 145 severe COVID-19 patients. Genetic association analysis was conducted between mild and severe COVID-19 patients, between all COVID-19 patients and general population, or between severe COVID-19 patients and general population. A total of 49 variants were confirmed to be associated with susceptibility or severity of COVID-19 (*p* < 0.05), corresponding to 18 independent loci. Specifically, rs1799964 in the promoter of inflammation-related gene *TNF*, rs9975538 in the intron of interferon receptor gene *IFNAR2*, rs429358 in the exon of *APOE*, rs1886814 in the intron of *FOXP4-AS1* and a list of variants in the widely reported 3p21.31 and *ABO* gene were confirmed. It is worth noting that, for the confirmed variants, the phenotypes of the cases and controls were highly consistent between our study and previous reports, and the confirmed variants identified between mild and severe patients were quite different from those identified between patients and general population, suggesting the genetic basis of susceptibility and severity of SARS-CoV-2 infection might be quite different. Moreover, we newly identified 67 significant associated variants in the 12 regulatory regions of 11 candidate genes (*p* < 0.05). Further annotation by RegulomeDB database and GTEx eQTL data filtered out two variants (rs11246060 and rs28655829) in the enhancer of broad-spectrum antiviral gene *IFITM3* that might affect disease severity by regulating the gene expression. Collectively, we confirmed a list of previously reported variants and identified novel regulatory variants associated with susceptibility and severity of COVID-19, which might provide biological and clinical insights into COVID-19 pathogenesis and treatment.

## Introduction

Coronavirus disease 2019 (COVID-19), an infectious disease caused by Severe Acute Respiratory Syndrome-Coronavirus 2 (SARS-CoV-2) (Zhu et al., 2020), has spread worldwide, resulting more than 460 million infections and six million deaths up to 16 March 2022 (https://covid19.who.int/). The occurrence and clinical outcomes of COVID-19 have been revealed great heterogeneity, ranging from insensitive, asymptomatic, mild, moderate to severe, critical or even death (Wu and McGoogan, 2020). Host factors such as age, gender, comorbidities were reported to be associated with this heterogeneity (Zhou et al., 2020a; Richardson et al., 2020).

Host genetic variants might also affect susceptibility and severity of coronavirus infection, as indicated by previous studies of SARS, Middle East Respiratory Syndrome (MERS) and emerging studies of COVID-19 (Di Maria et al., 2020). The first genome-wide association study (GWAS) of COVID-19 reported two severity-associated loci in Italians and Spanish: the 3p21.31 locus containing several immune genes and *ABO* locus determining *ABO* blood groups (Ellinghaus et al., 2020). The COVID-19 Host Genetics Initiative (HGI) was established to bring together global effort to elucidate the role of host genetic factors in susceptibility and severity of the SARS-CoV-2 virus pandemic (Initiative, 2020). However, current reported genetic studies of COVID-19 are mainly based on European populations. Whether these findings could apply to other populations was unknown.

Besides GWAS studies, several candidate gene studies indicated that certain variants in the type I interferon (IFN) pathway genes and SARS-CoV-2 receptor/coreceptor genes were associated with susceptibility and severity of COVID-19 (Zhang et al., 2020a; Benetti et al., 2020; Zhang et al., 2020b; Kuo et al., 2020; Latini et al., 2020; Novelli et al., 2020). For example, rs12252, variant of *IFITM3*, was reported to be associated with severe COVID-19 (Zhang et al., 2020b). Many rare loss-of-function variants in IFN-pathway genes such as *TLR3*, *IRF3*, *IRF7*, *IFNAR1,* and *IFNAR2* were identified to be associated with severe COVID-19 through impairing IFN immunity (Zhang et al., 2020a). Furthermore, in the *ACE2* and *TMPRSS2* genes, receptor and coreceptor gene for SARS-CoV-2 respectively, certain variants showed significantly different allele frequencies between COVID-19 patients and general population (Benetti et al., 2020; Latini et al., 2020; Novelli et al., 2020). However, these studies only focus on the genic region. The regulatory regions of these important genes are a lack of attention. Variants in regulatory regions, especially enhancers, could disrupt regulatory function, affect gene expression and thus contribute to susceptibility and severity of virus infection (Li et al., 2017; Downes et al., 2021). Therefore, it is necessary to pay more attention to variants in enhancers of important genes.

Here, we incorporated results of previous studies and designed a capture panel covering previously reported associated variants and regulatory regions of key genes. Using this panel, we targeted sequenced 96 mild and 145 severe COVID-19 patients. Genetic association analysis was conducted between mild and severe cases as well as ancestry-matched populations from 1000 Genomes Project. A list of previously reported associated variants was confirmed and two variants in the enhancers of *IFITM3* genes that might affect disease severity by regulating the gene expression were newly identified, which will lead to a better understanding of the host genetic factors at play in COVID-19.

## Materials and methods

### Study participants and recruitment

This study included 241 hospitalized COVID-19 patients recruited from Huoshenshan hospital at Wuhan city, Hubei province, China between 11 January 2020 and 11 March 2020. COVID-19 was diagnosed based on chest computed tomography (CT) manifestations and/or reverse transcription-polymerase chain reaction (RT-PCR) following the criteria of the New Coronavirus Pneumonia Prevention and Control Program (5th edition). In this study, the mild COVID-19 patients were those with no obvious clinical symptoms or with fever, respiratory symptoms, and radiological evidence of pneumonia. The severe COVID-19 patients were those having at least one of the following conditions: respiratory distress, respiratory rate ≤30 beats/minute; mean oxygen saturation ≤93% in a resting state; arterial blood oxygen partial pressure/oxygen concentration ≤300 mm·Hg; respiratory failure and requiring mechanical ventilation; shock; and admission to intensive care unit (ICU) with other organ function failure. Classifications of COVID-19 severity were taken as the worst classification during the patient’s hospital stay.

The clinical characteristics of the patients were extracted from the electronic medical records. We collected three broad classes of characteristics: 1) demographic variables (age, sex, and ethnicity); 2) symptoms (fever and diarrhea); and 3) comorbid conditions (hypertension, diabetes, cardiac disease, chronic bronchitis, chronic liver disease, chronic obstructive pulmonary disease, cerebrovascular disease, and cancer).

The Ethics Committee of Huoshenshan Hospital approved the study (HSSLL036). Given the urgency of the COVID-19 pandemic, the need for informed consent was waived by the ethnics boards of the hospital.

### Candidate variants and enhancers selection and probe design

We collected 30 unique variants associated with susceptibility or severity of SARS-CoV/SARS-CoV-2 infection from 21 papers (up to 29 November 2020), referred as “literature dataset”. In addition, we downloaded COVID19-hg GWAS meta-analysis results (release 4) produced by COVID-19 host genetics initiative (HGI) from https://www.covid19hg.org/results/r4/, and 1420 unique variants were selected that meet one of the following four requirements: 1) variants with *p* < 1E-5 in “A1_ALL” group, that is, phenotype of very severe respiratory confirmed covid vs. not hospitalized covid; 2) variants with *p* < 1E-5 in “B1_ALL” group, that is, phenotype of hospitalized covid vs. not hospitalized covid; 3) variants with *p* < 5E-7 in any one of group; 4) variants with *p* < 1E-5 in any three of groups except “A1_ALL” and “B1_ALL”. This was referred as “HGI dataset”. Two variants were overlapped in the two datasets, resulting in a total of 1448 unique variants collected.

IFNs play a central role in innate immunity against virus infection (Zhang et al., 2020a; Bastard et al., 2020) and cell receptors for virus are key determinant for viral entry (Hoffmann et al., 2020a; Shang et al., 2020). Therefore, We collected 19 IFN-pathway genes previously implicated in SARS-CoV/SARS-CoV-2 susceptibility and severity (Hamano et al., 2005; He et al., 2006; Ching et al., 2010; Zhang et al., 2020a; Zhang et al., 2020b) as well as 3 human cell receptors/co-receptors for SARS-CoV-2 (Hoffmann et al., 2020a; Hoffmann et al., 2020b; Zhou et al., 2020b; Shang et al., 2020). A total of 25 potential enhancers for these genes were obtained from GeneHancer database (GH score > 1, Gene Association > 100) (Fishilevich et al., 2017).

RNA probes were designed to cover these variants and gene enhancers. All the 25 enhancers and 1238 unique candidate variants were covered, including 29 variants in the literature dataset and 1211 variants in the HGI dataset (Supplementary Tables S1–S3).

### Targeted capture sequencing

Peripheral whole blood samples were collected from all participants. Genomic DNAs were extracted from 1 ml of peripheral whole blood, according to the manufacturer’s instructions (QIAamp DNA blood kits). The quality of the isolated genomic DNA was verified by the following two methods: 1) the DNA degradation and contamination were monitored in 1% agarose gels; and 2) the DNA concentration was measured using a Qubit DNA Assay Kit and a Qubit 3.0 Fluorometer (Life Technologies).

The targeted capture sequencing was conducted by iGeneTech Bioscience Corporation (Beijing, China). Briefly, Human genomic DNA was sheared to 150–200 bp by Bioruptor Pico (Diagenode). Then end repair, dA-tailing, and adapter ligation were performed. The ligation product was cleaned up and size-selected by using Beckman Ampure XP Beads (Beckman). The purified ligated product was amplified by using PCR. Then the library was in solution hybrid with biotinylated RNA probes, captured with Dynabeads MyOne Streptavidin T1 (Invitrogen), and amplified with PCR. The library was quantified by Qubit and fragment-size measured by Agilent 2100 Bioanalyzer system before high-throughput sequenced by NovaSeq.

### Variant calling and genetic association analysis

Raw reads were firstly quality trimmed with Trimmomatic (Bolger et al., 2014). Clean reads were then aligned to the human reference genome (hg38) using BWA algorithm (Li and Durbin, 2009). PCR duplicates were removed using samtools (Li et al., 2009), and GATK software (McKenna et al., 2010) was used to call SNPs and indels. The detected variants were finally saved as VCF files. Data of autosomal biallelic variants for Han Chinese of 1000 Genomes Project (Auton et al., 2015) were downloaded from https://www.internationalgenome.org/. Genetic association analysis was conducted using PLINK 1.9 software (Chang et al., 2015).

### Statistical analysis

*p*-values comparing demographics severe and mild disease groups were calculated by means of χ2 or Fisher exact test as appropriate, except for *p*-value for age which was calculated using student’s t test. Minor allele frequency (MAF) of variants was compared between case and control groups using Fisher exact test. Logistic regression analysis was also used to compute the contributing variants to severity with adjusting age, sex and comorbidities. Statistic power was calculated using G*Power 3 software (Faul et al., 2007). *p* < 0.05 was considered statistically significant.

## Results

### Clinical features of the COVID-19 patients

A total of 241 COVID-19 patients were recruited, with 96 mild cases and 145 severe cases. Demographic and phenotypic data are shown in Table 1. Comparing mild patients with severe ones, the median age increased from 58 to 67 years (*p* < 0.0001). In addition, we observed a greater percentage of severe patients with comorbidities than mild patients (*p* = 0.03), specifically with diabetes (*p* = 0.04) and cerebrovascular disease (*p* = 0.03). This is in accordance with previous report that older COVID-19 patients and those with comorbidities were more likely to be severe in disease (Zhou et al., 2020a; Richardson et al., 2020). Previous report also indicated that severe patients had a greater percentage of male cases (Richardson et al., 2020). However, no obvious sex difference was found between mild and severe patients in our data (*p* = 0.17).

**TABLE 1:** Patient demographics by clinical phenotype.

		Patients hospitalized with COVID-19, no. (%)^a^		
Characteristic		Mild (*N* = 96)	Severe (*N* = 145)	*p*-value*
Age, median (IQR)		58 (50.5–66.5)	67 (59–74)	<0.0001***
Male		45 (46.88)	81 (55.86)	0.17
Comorbidities		32 (35.45)	69 (50.83)	0.03*
	Hypertension	23 (22.73)	48 (32.04)	0.13
	Diabetes	9 (8.18)	28 (20.44)	0.04*
	Cardiac disease	5 (4.55)	13 (8.84)	0.33
	Chronic bronchitis	0 (0.91)	4 (2.76)	0.15
	COPD	0 (0)	2 (1.1)	0.52
	Chronic liver disease	3 (2.73)	3 (1.66)	0.68
	Cancer	1 (0.91)	6 (3.31)	0.25
	Cerebrovascular disease	1 (0.91)	11 (6.63)	0.03*
Presenting symptoms				
	Fever	71 (71.82)	106 (71.82)	0.88
	Diarrhea	1 (1.82)	7 (4.42)	0.15

^a^ Data represent no. (%) of patients unless otherwise specified.

*p-values comparing severe and mild disease groups were calculated by means of χ^2^ or Fisher exact tests as appropriate, except for *p*-value for age which was calculated using student’s *t* test. Abbreviations: IQR, interquartile range; COPD, chronic obstructive pulmonary disease.

### Targeted capture sequencing

**FIGURE 1:**
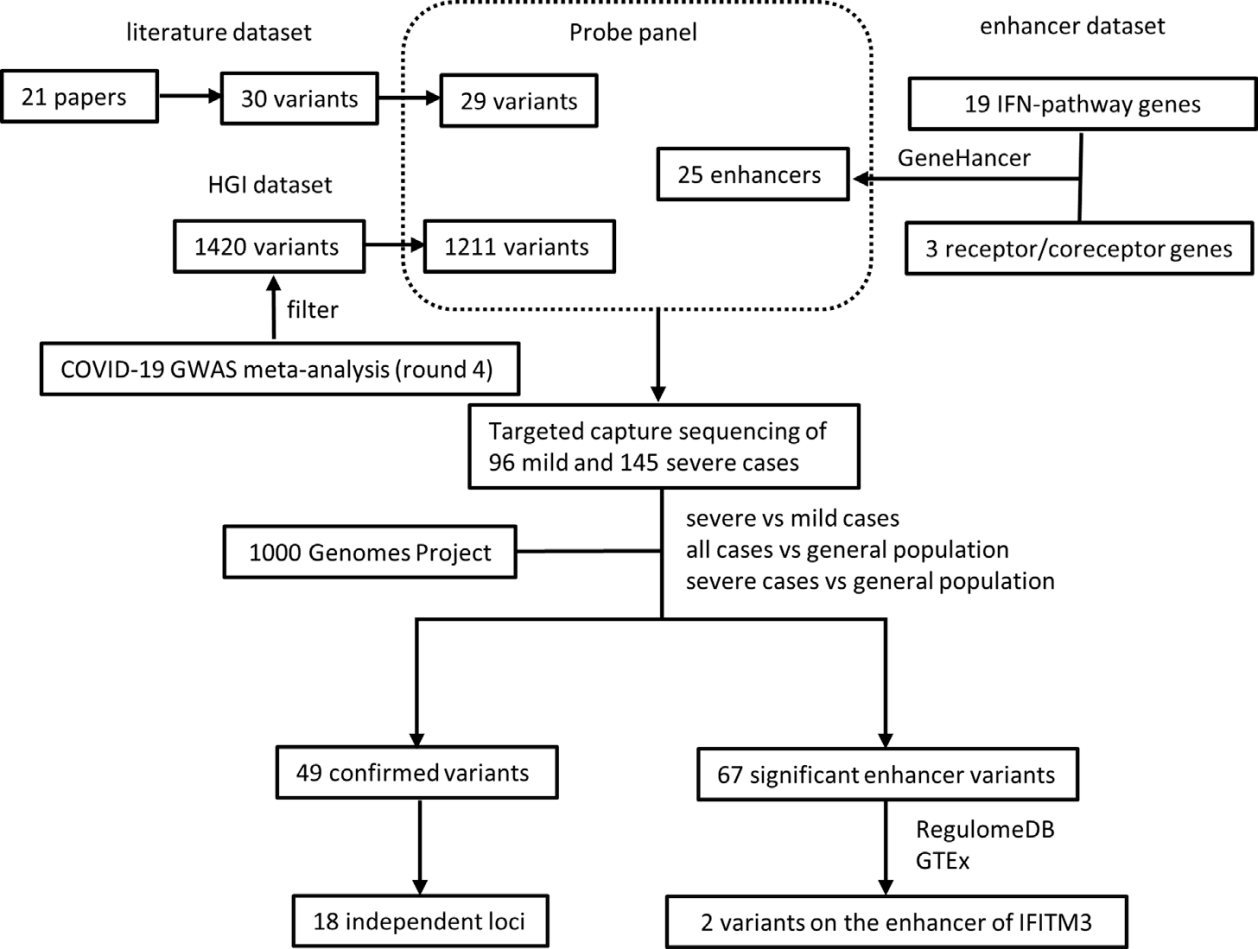
Illustration of experimental design and analysis.

The experimental design was illustrated in Figure 1. To validate previously reported variants associated with COVID-19 susceptibility and severity, we collected 30 unique variants from 21 papers (referred as “literature dataset”) and selected 1420 unique variants from COVID-19 host genetics initiative (HGI) release 4 (Initiative, 2020) (referred as “HGI dataset”). Additionally, to identify regulatory variants associated with COVID-19 susceptibility and severity in the enhancers of previously reported associated genes, we collected 19 IFN-pathway genes previously implicated in coronavirus susceptibility and severity (Hamano et al., 2005; He et al., 2006; Ching et al., 2010; Zhang et al., 2020a; Zhang et al., 2020b) as well as 3 human cell receptors/co-receptors for SARS-CoV-2 (Hoffmann et al., 2020a; Hoffmann et al., 2020b; Zhou et al., 2020b; Shang et al., 2020) and obtained 25 potential enhancers for these genes from GeneHancer database (Fishilevich et al., 2017) (referred as “enhancer dataset”).

A panel of RNA probes was designed to capture these variant and enhancers, resulting in 1238 unique variants and all the 25 potential enhancers covered (Supplementary Tables S1–S3). The 1238 unique variants included 29 variants in the literature dataset and 1211 variants in the HGI dataset, with two variants overlapped between the two datasets. Variants that fail to design probes might be due to GC content, repetitive sequence, dimer or secondary structure of probes.

The probe panel was used to targeted capture sequence the 241 COVID-19 patients. A median of 8.5 million raw reads were obtained for each sample. After filtering, a median of 8.3 million reads was kept as clean reads, with a median of average insert size of 173 bp. The reads were mapped to hg38 genome. The median mapping rate was 99.56%, with a median duplication rate of 27.47% (Supplementary Figure S1). Specifically, the median number of target mapped reads was 4.5 million, with a median target coverage rate of 99.57% and median target mean depth of 1472× (Supplementary Figure S2).

### Confirmation of genetic variants associated susceptibility and severity

Out of the 1238 candidate variants in the panel, a total of 1006 variants in the panel were identified in the 241 COVID-19 patients, including 26 variants in the literature dataset and 982 variants in the HGI dataset.

Comparing 96 mild and 145 severe COVID-19 patients, seven variants were confirmed to be significantly different in minor allele frequency (MAF) (*p* < 0.05, Supplementary Table S2), corresponding to four independent loci. The four lead variants were shown in Table 2. Specifically, rs1799964 in the promoter of inflammation-related gene *TNF* was found to be associated with COVID-19 severity. CC genotype of this variant has been reported to be associated with femoral head necrosis after SARS-CoV infection (Wang et al., 2008). In addition, rs2224986 and rs13062942 were still associated with COVID-19 severity after adjusting age, sex and comorbidities on regression analysis. Both variants had a significant difference between hospitalized and non-hospitalized patients in the HGI dataset.

**TABLE 2:** Independent lead variants that were confirmed between mild and severe cases.

SNP	Position	Allele (major/minor)	MAF (case/control)	*p*	OR (95%CI)	Mapped gene	Previous study	Previous study phenotype
rs13062942	3:62951091	A/G	0.51/0.39	1.17E-02	1.63 (1.12–2.35)	Intron of *LINC00698*	HGI	Hospitalized covid vs. not hospitalized covid
rs28373011	15:66669812	G/C	0.36/0.47	1.78E-02	0.63 (0.43–0.91)	Intron of *LINC01169*	HGI	Very severe respiratory confirmed covid vs. not hospitalized covid
rs1799964	6:31574531	T/C	0.15/0.23	2.92E-02	0.59 (0.37–0.93)	Upstream of *TNF*	PMID: 18312678	SARS cases with femoral head necrosis vs. SARS cases without femoral head necrosis
rs2224986	1:152712390	C/T	0.13/0.07	3.75E-02	1.98 (1.04–3.75)	Downstream of *LCE4A*	HGI	Hospitalized covid vs. not hospitalized covid

When comparing all COVID-19 patients with ancestry-matched general population from 1000 Genomes Project, we identified 39 significant variants (*p* < 0.05, Supplementary Table S5), corresponding 10 independent loci. The independent lead variants were shown in Table 3. Specifically, multiple genetic variants in the *ABO* gene locus and 3p21.31 region have been validated. In addition, the missense variant rs429358 in exon of *APOE* gene, which has been reported to be associated with COVID-19-positive in the UK Biobank data (Kuo et al., 2020), was confirmed in Chinese population. As for the confirmed variant rs1886814 in the intron of *FOXP4-AS1* (forkhead box P4 antisense RNA 1), recent trans-ethnic genome-wide association study of severe COVID-19 that incorporated Chinese population and HGI results also revealed a significant variant nearby, rs1853837 (Wu et al., 2021), which is LD with rs1886814 (1000 Genomes Project CHB, *r* ^2^ = 0.68).

**TABLE 3:** Independent lead variants that were confirmed between all COVID-19 patients and general population.

SNP	Position	Allele (major/minor)	MAF (case/control)	*p*	OR (95%CI)	Mapped gene	Previous study	Previous study phenotype
rs1291122587	9:133270497	GA/G	0.26/0.59	6.68E-24	0.24 (0.18–0.32)	Intron of *ABO*	HGI	Hospitalized covid vs. population
								Hospitalized covid vs. population, leave out 23andMe (EUR)
								Covid vs. population
								Covid vs. population, leave out 23andMe (EUR)
rs429358	19:44908684	T/C	0.1/0	6.67E-15	NA	Missense of *APOE*	PMID: 32451547	Positive COVID-19 patients vs. participants negative or not tested
rs71327056	3:46348485	C/G	0.01/0.04	2.74E-03	0.21 (0.07–0.64)	Intergenic of *UQCRC2P1-CCR2*	HGI	Very severe respiratory confirmed covid vs. population
								Hospitalized covid vs. population
								Hospitalized covid vs. population, leave out 23andMe (EUR)
rs9411475	9:133251881	T/C	0.33/0.26	1.08E-02	1.46 (1.09–1.95)	Downstream of *ABO*	HGI	Covid vs. population
rs495828	9:133279294	G/T	0.28/0.2	1.27E-02	1.49 (1.09–2.03)	Upstream of *ABO*	HGI	Hospitalized covid vs. population
								Covid vs. lab/self-reported negative
								Covid vs. population
								Very severe respiratory confirmed covid vs. population
								Hospitalized covid vs. population, leave out 23andMe (EUR)
rs10734222	11:14244913	G/A	0.18/0.24	2.19E-02	0.68 (0.49–0.94)	Intron of *SPON1*	HGI	Hospitalized covid vs. population, leave out 23andMe (EUR)
rs1886814	6:41534945	A/C	0.45/0.37	2.55E-02	1.36 (1.04–1.77)	Intron of *FOXP4-AS1*	HGI	Hospitalized covid vs. population
rs973579	19:48738719	A/G	0/0.02	2.88E-02	0.12 (0.02–1.01)	Upstream of *RASIP1*	HGI	Covid vs. lab/self-reported negative
rs74609750	21:33896073	C/A	0.3/0.23	2.93E-02	1.40 (1.04–1.89)	3′ UTR of *ITSN1*	HGI	Covid vs. population
rs898467998	9:133260743	G/A	0.27/0.34	3.58E-02	0.74 (0.55–0.98)	Intron of *ABO*	HGI	Covid vs. lab/self-reported negative
								Covid vs. population
								Hospitalized covid vs. population

When comparing severe COVID-19 patients with ancestry-matched general population from 1000 Genomes Project, we identified 23 significant variants (*p* < 0.05, Supplementary Table S6), corresponding 7 independent loci. The independent lead variants were shown in Table 4. Again, multiple genetic variants in the *ABO* gene locus and 3p21.31 region, rs429358 in exon of *APOE* gene and rs1886814 in the intron of *FOXP4-AS1* have been validated. What’s more, rs9975538 in the intron of gene *IFNAR2* was also confirmed. *IFNAR2* gene, along with *IFNAR1* encodes type I interferon receptor, thus the rs9975538 might affect type I interferon pathway.

**TABLE 4:** Independent lead variants that were confirmed between severe COVID-19 patients and general population.

SNP	Position	Allele (major/minor)	MAF (case/control)	*p*	OR (95%CI)	Mapped gene	Previous study	Previous study phenotype
rs1291122587	9:133270497	GA/G	0.26/0.59	7.02E-18	0.24 (0.18–0.34)	Intron of *ABO*	HGI	Hospitalized covid vs. population
								Hospitalized covid vs. population, leave out 23 and Me (EUR)
								Covid vs. population
								Covid vs. population, leave out 23 and Me (EUR)
rs8111981	19:977763	G/A	0.28/0.35	3.37E-02	0.70 (0.50–0.97)	Downstream of *ARID3A*	HGI	Very severe respiratory confirmed covid vs. population
rs9975538	21:33254549	C/T	0.29/0.37	4.37E-02	0.71 (0.52–0.98)	Intron of *IFNAR2*	HGI	Very severe respiratory confirmed covid vs. population
								Hospitalized covid vs. population
								Hospitalized covid vs. population, leave out 23 and Me (EUR)
rs429358	19:44908684	T/C	0.11/0	4.29E-14	NA	Missense of *APOE*	PMID: 32451547	Positive COVID-19 patients vs. participants negative or not tested
rs10734222	11:14244913	G/A	0.17/0.24	1.19E-02	0.61 (0.42–0.90)	Intron of *SPON1*	HGI	Hospitalized covid vs. population, leave out 23 and Me (EUR)
rs59492037	6:4571951	A/G	0.27/0.35	2.63E-02	0.68 (0.49–0.95)	Intron of *AL162718.1*	HGI	Very severe respiratory confirmed covid vs. not hospitalized covid
rs4302292	21:33919239	G/A	0.31/0.23	3.07E-02	1.47 (1.05–2.05)	Upstream of *ATP5PO*	HGI	Covid vs. population
rs71327056	3:46348485	C/G	0.01/0.04	3.12E-02	0.27 (0.08–0.92)	Intergenic of *UQCRC2P1-CCR2*	HGI	Very severe respiratory confirmed covid vs. population
								Hospitalized covid vs. population
								Hospitalized covid vs. population, leave out 23 and Me (EUR)
rs1886814	6:41534945	A/C	0.45/0.37	3.60E-02	1.39 (1.03–1.88)	Intron of *FOXP4-AS1*	HGI	Hospitalized covid vs. population

In total, 49 unique variants were confirmed to be associated with susceptibility or severity of COVID-19 (*p* < 0.05), corresponding 18 independent loci. It is worth noting that, for the confirmed variants, the phenotypes of the cases and controls were highly consistent between our study and previous reports, and the confirmed variants identified between mild and severe patients were quite different from those identified between patients and general population, suggesting the genetic basis of susceptibility and severity of SARS-CoV-2 infection might be quite different.

### Identification of associated genetic variants in the enhancers of key genes

Previous candidate gene study of genetic association with COVID-19 mainly focused on the exon region of important genes (Zhang et al., 2020a; Novelli et al., 2020). However, regulatory regions, particularly enhancers, play a vital role in gene expression and may affect disease susceptibility and severity when misfunction (Claringbould and Zaugg, 2021). To investigate the role of enhancer variants of key genes in COVID-19, we also included 25 enhancers of 16 IFN-pathway genes and 3 SARS-CoV-2 receptor/co-receptor genes in the probe panel. These enhancers were predicted by GeneHancer database. Genetic association analysis was conducted between mild and severe COVID-19 patients, between all COVID-19 patients and ancestry-matched general population from 1000 Genomes Project, or between severe COVID-19 patients and ancestry-matched general population from 1000 Genomes Project.

In total, we identified 67 variants in the enhancer region of the panel that were associated with susceptibility or severity of COVID-19, relating to 12 enhancers of 11 genes (*p* < 0.05, Supplementary Tables S7–S9). Further annotation by RegulomeDB database filtered out five potential regulatory variants for broad-spectrum antiviral gene *IFITM3* and one for SARS-CoV-2 co-receptor gene *TMPRSS2* (probability score > 0.8, Table 5). Specifically, among the six variants, GTEx revealed two variants affecting the expression of *IFITM3*, that is, T allele of rs11246060 in the enhancer of *IFITM3*, which protected COVID-19 patients from severe outcomes when comparing mild and severe patients (*p* = 2.38E-2, OR = 0.39, 95% CI = 0.17–0.89), was associated with increased expression of *IFITM3* in whole blood (*p* = 1.72E-10), while T allele of rs28655829 in the enhancer of *IFITM3*, which increased the risk of severity when comparing mild and severe patients (*p* = 2.38E-2, OR = 0.39, 95% CI = 0.17–0.89), was associated with decreased expression of *IFITM3* in cultured fibroblasts (*p* = 7.55E-8). This indicated that these variants might confer genetic risk or protection by affecting the gene expression and highlighted the importance of *IFITM3* gene in the defense of SARS-CoV-2 infection.

**TABLE 5:** Significant enhancer variants that were associated with susceptibility and severity of COVID-19 and predicted regulatory by RegulomeDB.

Enhancer	Gene	SNP	Position	Allele (major/minor)	MAF (case/control)	*p*	OR (95%CI)	Phenotype	RegulomeDB probability
11:304431-322801	*IFITM3*	rs11246060	11:306877	C/T	0.03/0.08	2.38E-02	0.39 (0.17–0.89)	Severe covid vs. mild covid	0.9224
11:304431-322801	*IFITM3*	rs11246060	11:306877	C/T	0.05/0.03	4.27E-02	2.13 (1.04–4.37)	All covid vs. population	0.9224
11:304431-322801	*IFITM3*	rs79196191	11:316475	G/T	0.07/0.03	8.33E-03	2.43 (1.24–4.78)	All covid vs. population	0.99633
11:304431-322801	*IFITM3*	rs79196191	11:316475	G/T	0.08/0.03	2.52E-03	2.94 (1.44–6.01)	Severe covid vs. population	0.99633
11:304431-322801	*IFITM3*	rs28655829	11:320166	C/T	0.05/0.01	3.44E-02	4.82 (1.08–21.45)	Severe covid vs. mild covid	0.8658
11:304431-322801	*IFITM3*	rs1248936100	11:321074	T/TG	0.03/0.08	1.52E-02	0.33 (0.14–0.81)	Severe covid vs. mild covid	0.9975
11:324001-331788	*IFITM3*	rs10902125	11:329951	A/G	0.04/0.01	3.42E-02	4.46 (0.99–19.98)	Severe covid vs. mild covid	0.96307
21:41496486-41511100	*TMPRSS2*	rs1440999733	21:41501641	C/A	0/0.02	2.47E-02	0.00 (0.00-NaN)	Severe covid vs. mild covid	0.85643

## Discussion

In this study, out of 1238 variant previously reported to be association with susceptibility and severity of COVID-19, we confirmed 49 variants, corresponding to 18 independent loci, including 3p21.31 locus, *ABO*, *IFNAR2*, *TNF*, *APOE*, and *FOXP4-AS1* gene.

3p21.31 locus has been identified as a risk factor by GWAS studies of Italian and Spanish (Ellinghaus et al., 2020), British (Wang et al., 2008), Americans (Shelton et al., 2021), and meta-GWAS study of HGI (Initiative, 2021). However, most participants of the studies are Europeans and the major genetic risk factor in 3p21.31 for severe COVID-19 is proposed to be inherited from Neanderthals (Zeberg and Pääbo, 2020), which is almost absent in East Asians. In accordance, previous COVID-19 GWAS study of Chinese population was unable to replicate the locus (Wang et al., 2020; Wu et al., 2021). In our study, consistent with the low frequency of the previously reported lead variant rs11385942 in 3p21.31, only one individual was identified to harbor this variant. This individual had severe COVID-19, which might be due to the risk variant rs11385942. On the other hand, we confirmed another variant in 3p21.31, rs71327056, which is an intergenic variant between *UQCRC2P1* and *CCR2* genes and is not linkage disequilibrium (LD) with rs11385942 (Ellinghaus et al., 2020) (1000 Genomes Project CEU, *r* ^2^ = 0.19), suggesting there might be more than one independent variant in 3p21.31 contribution to COVID-19 susceptibility and severity. Notably, the minor allele G of rs71317056 was found to increase severity in HGI release4 where most of individuals were Europeans while our study revealed that the minor allele of rs71317056 effected oppositely in Chinese population (Tables 3, 4). We speculated this might due to the interaction with another risk haplotype which Europeans inherited from Neanderthals but was absent in Asians. In addition, rs71317056 is in LD with rs35943069 (1000 Genomes Project CEU, *r* ^2^ = 1; CHB, *r* ^2^ = 1), which resides in a potentially enhancer region that is annotated by GeneHancer (Fishilevich et al., 2017). The minor allele is associated with increased *CCR1* gene expression in cultured fibroblasts (GTEx V8, Supplementary Table S10) (Consortium, 2020), suggesting that it might function through expression regulation of *CCR1*, receptor for a C-C type chemokine which play an important role in immune system against viral infection (Zlotnik and Yoshie, 2012).

The *ABO* locus has also been revealed to be associated with COVID-19 severity (Ellinghaus et al., 2020). However, the previously reported lead variant rs657152 had no significant difference in allele frequency between cases and controls of our study. Instead, we confirmed several other variants in the *ABO* locus when comparing all COVID-19 cases with general Chinese population. Specifically, one of the lead variants, rs34357864 was also been confirmed when comparing severe COVID-19 cases with general Chinese population. Similar to our study, HGI data revealed that this variant was significant when either all COVID-19 patients or hospitalized COVID-19 patients compared with general population, suggesting this is a variant associated with susceptibility.

The T allele of rs9975538 in the intron of gene *IFNAR2* had a lower frequency in severe COVID-19 patients compared with general Chinese population, consistent with results of HGI release 4 comparing either very severe respiratory confirmed COVID-19 patients or hospitalized COVID-19 patients with general population. rs9975538 was also in LD with rs2236757 (1000 Genomes Project CEU, *r* ^2^ = 0.75; CHB, *r* ^2^ = 1), the previously reported variant that was associated with critical illness of COVID-19 (Pairo-Castineira et al., 2021). Notably, the T allele of rs9975538 increased the expression of *IFNAR2* and *IL10RB* in lung (GTEx V8, *p* = 1.09E-5, *p* = 1.99E-5 respectively). As *IFNAR2* and *IL10RB* are type I and III IFN receptor respectively, this variant might confer protection by increasing IFN receptor expression and thus upregulating the antiviral activity of IFN pathway. In addition, *IFNAR2* play a vital role in multiple sclerosis, a chronic autoimmune disorder characterized by inflammation of the central nervous system, demyelination and axonal damage (Gilli et al., 2008; Órpez-Zafra et al., 2017). This leads to a hypothesis that COVID-19 patients harboring *IFNAR2* rs9975538 variant might be more likely to develop neurological disorders (Douaud et al., 2022; Lee et al., 2022), possibly though neuroinflammatory pathways.

Rs1799964, which is located upstream of *TNF* gene, was found to be associated with disease severity, with C allele being associated with mild phenotype. TNF is multifunctional proinflammatory cytokine and plays a key role in regulating the immunological response to infections (Waters et al., 2013). The C allele of rs1799964 was associated with increased expression of *TNF* than T allele (Nourian et al., 2017) and increased lymphocyte counts (Chen et al., 2020), which might protect the body from severe disease.

*APOE* gene polymorphisms have been reported to be associated with susceptibility or severity of COVID-19 in British, Czech, Spanish, Finnish and Kurdish population (Kuo et al., 2020; Al-Jaf et al., 2021; Del Ser et al., 2021; Hubacek et al., 2021; Kurki et al., 2021). Consistent with above findings, we revealed an association of *APOE* variant rs429358 with susceptibility to COVID-19 in Chinese population. Given that *APOE* is associated with Alzheimer’s and cardiovascular diseases and type 2 diabetes (Liu et al., 2013; Mahley, 2016; Liu et al., 2019), comorbidities that are related to COVID-19 susceptibility and severity, the effect of the *APOE* variant on COVID-19 could be indirect. Meanwhile, recent researches indicated that APOE might also affect SARS-CoV-2 infection directly by interacting with ACE2 inhibiting SARS-CoV-2 cellular entry (Zhang et al., 2022), regulating cellular cholesterol homeostasis (Gao et al., 2022) and modulating antiviral immunity (Ostendorf et al., 2022). Notably, another variant that determines APOE isoforms, rs7412, did not pass the significant threshold, probably because this variant mainly contributes to APOE ε2 isoform while COVID-19 is more associated with APOE ε4 isoform (Kuo et al., 2020; Al-Jaf et al., 2021; Del Ser et al., 2021; Hubacek et al., 2021; Kurki et al., 2021).

Rs1886814 in the intron of the lncRNA *FOXP4-AS1* was found to be associated with disease susceptibility when comparing all COVID-19 patients or severe COVID-19 patients with general Chinese population. In HGI release 4 datasets, it is associated with COVID-19 hospitalization when comparing with general population. Recent trans-ethnic genome-wide association study of severe COVID-19 that incorporated Chinese population and HGI results revealed another significant variant in the intron of *FOXP4-AS1*, rs1853837, which is LD with rs1886814 in Chinese population (1000 Genomes Project CHB, *r* ^2^ = 0.68) (Wu et al., 2021). The risk allele C of rs1886814 is an eQTL in positive association with the expression of FOXP4 in lung (GETx V8, *p* = 3.28E-6) (Consortium, 2020). FOXP4 is a transcription factor expressed in both thymocytes and peripheral CD4^+^ and CD8^+^ T cells, and is necessary for normal T cell cytokine recall responses to antigen following pathogenic infection (Wiehagen et al., 2012).

We noted that the overall confirmation rate was not high, possibly due to different population structure and limited sample size of our study. The statistic power was provided in Supplementary Figure S3. On the other hand, it is worth noting that for the validated variants, the phenotypes of cases and controls were highly coordinated in our study and original study. All variants validated in our mild and severe group were specific to be identified in previous association study of severity, that is, when comparing hospitalized or very severe respiratory confirmed COVID-19 patients with not hospitalized ones. Nearly all variants validated in the COVID-19 patients and general Chinese population group were specific to be identified in previous association study of susceptibility, that is, comparing COVID-19 patients with general population. The remarkable specificity suggested that susceptibility and severity might have different genetic basis and also indicated the accuracy of our study.

In addition, we also identified 67 variants in the 12 regulatory regions of 11 candidate genes associated with susceptibility or severity of COVID-19, which have not been reported before. Further annotation by RegulomeDB and GTEx database revealed two variants affected the expression of *IFITM3* and conferred genetic risk and protection respectively. *IFITM3* gene encodes a transmembrane protein that could be induced by interferons and function as a broad-spectrum antiviral effector molecule by directly limiting cellular entry of a number of pathogenic viruses, including influenza A virus, West Nile virus, dengue virus, SARS-CoV and SARS-CoV-2 (Diamond and Farzan, 2013; Shi et al., 2021). Moreover, rs12252 variant in the gene has been found to be associated with COVID-19 severity (Zhang et al., 2020b; Alghamdi et al., 2021; Gómez et al., 2021). Our results indicated that, in addition to genetic variants, enhancer variants of *IFITM3* might confer genetic risk or protection by affecting gene expression as well. Though larger cohort studies are needed to confirm these genetic associations, our data presented here also highlighted the important role of *IFITM3* in host defense against SARS-CoV-2.

In conclusion, we confirmed a list of previously reported variants associated with susceptibility and severity of COVID-19, and identified several enhancer variants potentially regulating expression of genes associated with COVID-19. Though larger cohort studies and further experiments are needed to confirm these genetic associations and explore the molecular mechanism, elucidation of host genetic factors contributing to susceptibility to severe infection will provide the opportunity for clinical risk profiling of patients with COVID-19, mechanistic understanding of the underlying pathophysiology and further identification of potential therapeutic targets.

## Data Availability

The datasets presented in this study can be found in online repositories. The names of the repository/repositories and accession number(s) can be found in the article/Supplementary Material.

## References

[B1] Al-Jaf S. M. A. Niranji S. S. Ali H. N. Mohammed O. A. (2021). Association of Apolipoprotein e polymorphism with SARS-CoV-2 infection. Infect. Genet. Evol. 95, 105043. 10.1016/j.meegid.2021.105043 34419671 PMC8375275

[B2] Alghamdi J. Alaamery M. Barhoumi T. Rashid M. Alajmi H. Aljasser N. (2021). Interferon-induced transmembrane protein-3 genetic variant rs12252 is associated with COVID-19 mortality. Genomics 113 (4), 1733–1741. 10.1016/j.ygeno.2021.04.002 33838280 PMC8025598

[B3] Auton A. Brooks L. D. Durbin R. M. Garrison E. P. Kang H. M. Korbel J. O. (2015). A global reference for human genetic variation. Nature 526 (7571), 68–74. 10.1038/nature15393 26432245 PMC4750478

[B4] Bastard P. Rosen L. B. Zhang Q. Michailidis E. Hoffmann H-H. Zhang Y. (2020). Autoantibodies against type I IFNs in patients with life-threatening COVID-19. Science 370 (6515), eabd4585. 10.1126/science.abd4585 32972996 PMC7857397

[B5] Benetti E. Tita R. Spiga O. Ciolfi A. Birolo G. Bruselles A. (2020). ACE2 gene variants may underlie interindividual variability and susceptibility to COVID-19 in the Italian population. Eur. J. Hum. Genet. 28 (11), 1602–1614. 10.1038/s41431-020-0691-z 32681121 PMC7366459

[B6] Bolger A. M. Lohse M. Usadel B. (2014). Trimmomatic: A flexible trimmer for illumina sequence data. Bioinformatics 30 (15), 2114–2120. 10.1093/bioinformatics/btu170 24695404 PMC4103590

[B7] Chang C. C. Chow C. C. Tellier L. C. Vattikuti S. Purcell S. M. Lee J. J. (2015). Second-generation PLINK: Rising to the challenge of larger and richer datasets. Gigascience 4, 7. 10.1186/s13742-015-0047-8 25722852 PMC4342193

[B8] Chen M-H. Raffield L. M. Mousas A. Sakaue S. Huffman J. E. Moscati A. (2020). Trans-ethnic and ancestry-specific blood-cell genetics in 746, 667 individuals from 5 global populations. Cell 182 (5), 1198–1213. 10.1016/j.cell.2020.06.045 32888493 PMC7480402

[B9] Ching J. C-Y. Chan K. Y. K. Lee E. H. L. Xu M-S. Ting C. K. P. So T. M. K. (2010). Significance of the myxovirus resistance A (MxA) gene -123C>a single-nucleotide polymorphism in suppressed interferon beta induction of severe acute respiratory syndrome coronavirus infection. J. Infect. Dis. 201 (12), 1899–1908. 10.1086/652799 20462354 PMC7109798

[B10] Claringbould A. Zaugg J. B. (2021). Enhancers in disease: Molecular basis and emerging treatment strategies. Trends Mol. Med. 27 (11), 1060–1073. 10.1016/j.molmed.2021.07.012 34420874

[B11] Consortium G. T. (2020). The GTEx Consortium atlas of genetic regulatory effects across human tissues. Science 369 (6509), 1318–1330. 10.1126/science.aaz1776 32913098 PMC7737656

[B12] Del Ser T. Fernández-Blázquez M. A. Valentí M. Zea-Sevilla M. A. Frades B. Alfayate E. (2021). Residence, clinical features, and genetic risk factors associated with symptoms of COVID-19 in a cohort of older people in madrid. Gerontology 67 (3), 281–289. 10.1159/000513182 33429394 PMC7900450

[B13] Di Maria E. Latini A. Borgiani P. Novelli G. (2020). Genetic variants of the human host influencing the coronavirus-associated phenotypes (SARS, MERS and COVID-19): Rapid systematic review and field synopsis. Hum. Genomics 14 (1), 30. 10.1186/s40246-020-00280-6 32917282 PMC7484929

[B14] Diamond M. S. Farzan M. (2013). The broad-spectrum antiviral functions of IFIT and IFITM proteins. Nat. Rev. Immunol. 13 (1), 46–57. 10.1038/nri3344 23237964 PMC3773942

[B15] Douaud G. Lee S. Alfaro-Almagro F. Arthofer C. Wang C. McCarthy P. (2022). SARS-CoV-2 is associated with changes in brain structure in UK Biobank. Nature 604 (7907), 697–707. 10.1038/s41586-022-04569-5 35255491 PMC9046077

[B16] Downes D. J. Cross A. R. Hua P. Roberts N. Schwessinger R. Cutler A. J. (2021). Identification of LZTFL1 as a candidate effector gene at a COVID-19 risk locus. Nat. Genet. 53 (11), 1606–1615. 10.1038/s41588-021-00955-3 34737427 PMC7611960

[B17] Ellinghaus D. Degenhardt F. Bujanda L. Buti M. Albillos A. Invernizzi P. (2020). Genomewide association study of severe covid-19 with respiratory failure. N. Engl. J. Med. 383 (16), 1522–1534. 10.1056/NEJMoa2020283 32558485 PMC7315890

[B18] Faul F. Erdfelder E. Lang A-G. Buchner A. G. (2007). G*Power 3: A flexible statistical power analysis program for the social, behavioral, and biomedical sciences. Behav. Res. Methods 39 (2), 175–191. 10.3758/bf03193146 17695343

[B19] Fishilevich S. Nudel R. Rappaport N. Hadar R. Plaschkes I. Iny Stein T. (2017). GeneHancer: Genome-wide integration of enhancers and target genes in GeneCards. Oxford): Database. 10.1093/database/bax028 PMC5467550 28605766

[B20] Gao P. Ji M. Liu X. Chen X. Liu H. Li S. (2022). Apolipoprotein E mediates cell resistance to influenza virus infection. Sci. Adv. 8 (38), eabm6668. 10.1126/sciadv.abm6668 36129973 PMC9491715

[B21] Gilli F. Valentino P. Caldano M. Granieri L. Capobianco M. Malucchi S. (2008). Expression and regulation of IFNalpha/beta receptor in IFNbeta-treated patients with multiple sclerosis. Neurology 71 (24), 1940–1947. 10.1212/01.wnl.0000327340.50284.8d 18971450

[B22] Gómez J. Albaiceta G. M. Cuesta-Llavona E. García-Clemente M. López-Larrea C. Amado-Rodríguez L. (2021). The Interferon-induced transmembrane protein 3 gene (IFITM3) rs12252 C variant is associated with COVID-19. Cytokine 137, 155354. 10.1016/j.cyto.2020.155354 33113474

[B23] Hamano E. Hijikata M. Itoyama S. Quy T. Phi N. C. Long H. T. (2005). Polymorphisms of interferon-inducible genes OAS-1 and MxA associated with SARS in the Vietnamese population. Biochem. Biophys. Res. Commun. 329 (4), 1234–1239. 10.1016/j.bbrc.2005.02.101 15766558 PMC7092916

[B24] He J. Feng D. de Vlas S. J. Wang H. Fontanet A. Zhang P. (2006). Association of SARS susceptibility with single nucleic acid polymorphisms of OAS1 and MxA genes: A case-control study. BMC Infect. Dis. 6, 106. 10.1186/1471-2334-6-106 16824203 PMC1550407

[B25] Hoffmann M. Kleine-Weber H. Pöhlmann S. (2020). A multibasic cleavage site in the spike protein of SARS-CoV-2 is essential for infection of human lung cells. Mol. Cell 78 (4), 779–784. 10.1016/j.molcel.2020.04.022 32362314 PMC7194065

[B26] Hoffmann M. Kleine-Weber H. Schroeder S. Krüger N. Herrler T. Erichsen S. (2020). SARS-CoV-2 cell entry depends on ACE2 and TMPRSS2 and is blocked by a clinically proven protease inhibitor. Cell 181 (2), 271–280. 10.1016/j.cell.2020.02.052 32142651 PMC7102627

[B27] Hubacek J. A. Dlouha L. Dusek L. Majek O. Adamkova V. (2021). Apolipoprotein E4 allele in subjects with COVID-19. Gerontology 67 (3), 320–322. 10.1159/000516200 33965962 PMC8247822

[B28] Initiative C-H. G. (2021). Mapping the human genetic architecture of COVID-19. Nature 600 (7889), 472–477. 34237774 10.1038/s41586-021-03767-x PMC8674144

[B29] Initiative C-H. G. (2020). The COVID-19 Host Genetics Initiative, a global initiative to elucidate the role of host genetic factors in susceptibility and severity of the SARS-CoV-2 virus pandemic. Eur. J. Hum. Genet. 28 (6), 715–718. 10.1038/s41431-020-0636-6 32404885 PMC7220587

[B30] Kuo C-L. Pilling L. C. Atkins J. L. Masoli J. A. H. Delgado J. Kuchel G. A. (2020). APOE e4 Genotype Predicts Severe COVID-19 in the UK Biobank Community Cohort. J. Gerontol. A Biol. Sci. Med. Sci. 75 (11), 2231–2232. 10.1093/gerona/glaa131 32451547 PMC7314139

[B31] Kurki S. N. Kantonen J. Kaivola K. Hokkanen L. Mäyränpää M. I. Puttonen H. (2021). APOE ε4 associates with increased risk of severe COVID-19, cerebral microhaemorrhages and post-COVID mental fatigue: A Finnish biobank, autopsy and clinical study. Acta Neuropathol. Commun. 9 (1), 199. 10.1186/s40478-021-01302-7 34949230 PMC8696243

[B32] Latini A. Agolini E. Novelli A. Borgiani P. Giannini R. Gravina P. (2020). COVID-19 and genetic variants of protein involved in the SARS-CoV-2 entry into the host cells. Genes 11 (9), E1010. 10.3390/genes11091010 PMC7565048 32867305

[B33] Lee M. H. Perl D. P. Steiner J. Pasternack N. Li W. Maric D. (2022). Neurovascular injury with complement activation and inflammation in COVID-19. Brain. 145 (7), 2555–2568. 10.1093/brain/awac151 35788639 PMC9278212

[B34] Li H. Durbin R. (2009). Fast and accurate short read alignment with Burrows-Wheeler transform. Bioinformatics 25 (14), 1754–1760. 10.1093/bioinformatics/btp324 19451168 PMC2705234

[B35] Li H. Handsaker B. Wysoker A. Fennell T. Ruan J. Homer N. (2009). The sequence alignment/map format and SAMtools. Bioinformatics 25 (16), 2078–2079. 10.1093/bioinformatics/btp352 19505943 PMC2723002

[B36] Li P. Shi M-L. Shen W-L. Zhang Z. Xie D-J. Zhang X-Y. (2017). Coordinated regulation of IFITM1, 2 and 3 genes by an IFN-responsive enhancer through long-range chromatin interactions. Biochim. Biophys. Acta. Gene Regul. Mech. 1860 (8), 885–893. 10.1016/j.bbagrm.2017.05.003 28511927 PMC7102783

[B37] Liu C-C. Liu C-C. Kanekiyo T. Xu H. Bu G. (2013). Apolipoprotein E and alzheimer disease: Risk, mechanisms and therapy. Nat. Rev. Neurol. 9 (2), 106–118. 10.1038/nrneurol.2012.263 23296339 PMC3726719

[B38] Liu S. Liu J. Weng R. Gu X. Zhong Z. (2019). Apolipoprotein E gene polymorphism and the risk of cardiovascular disease and type 2 diabetes. BMC Cardiovasc. Disord. 19 (1), 213. 10.1186/s12872-019-1194-0 31521122 PMC6744677

[B39] Mahley R. W. (2016). Apolipoprotein E: From cardiovascular disease to neurodegenerative disorders. J. Mol. Med. 94 (7), 739–746. 10.1007/s00109-016-1427-y 27277824 PMC4921111

[B40] McKenna A. Hanna M. Banks E. Sivachenko A. Cibulskis K. Kernytsky A. (2010). The genome analysis toolkit: A MapReduce framework for analyzing next-generation DNA sequencing data. Genome Res. 20 (9), 1297–1303. 10.1101/gr.107524.110 20644199 PMC2928508

[B41] Nourian M. Chaleshi V. Pishkar L. Azimzadeh P. Baradaran Ghavami S. Balaii H. (2017). Evaluation of tumor necrosis factor (TNF)-α mRNA expression level and the rs1799964 polymorphism of the TNF-α gene in peripheral mononuclear cells of patients with inflammatory bowel diseases. Biomed. Rep. 6 (6), 698–702. 10.3892/br.2017.908 28584644 PMC5449959

[B42] Novelli A. Biancolella M. Borgiani P. Cocciadiferro D. Colona V. L. D'Apice M. R. (2020). Analysis of ACE2 genetic variants in 131 Italian SARS-CoV-2-positive patients. Hum. Genomics 14 (1), 29. 10.1186/s40246-020-00279-z 32917283 PMC7483483

[B43] Órpez-Zafra T. Pavía J. Hurtado-Guerrero I. Pinto-Medel M. J. Rodriguez Bada J. L. Urbaneja P. (2017). Decreased soluble IFN-β receptor (sIFNAR2) in multiple sclerosis patients: A potential serum diagnostic biomarker. Mult. Scler. 23 (7), 937–945. 10.1177/1352458516667564 27613121

[B44] Ostendorf B. N. Patel M. A. Bilanovic J. Hoffmann H. H. Carrasco S. E. Rice C. M. (2022). Common human genetic variants of APOE impact murine COVID-19 mortality. Nature 611, 346–351. 10.1038/s41586-022-05344-2 36130725 PMC10957240

[B45] Pairo-Castineira E. Clohisey S. Klaric L. Bretherick A. D. Rawlik K. Pasko D. (2021). Genetic mechanisms of critical illness in COVID-19. Nature 591 (7848), 92–98. 10.1038/s41586-020-03065-y 33307546

[B46] Richardson S. Hirsch J. S. Narasimhan M. Crawford J. M. McGinn T. Davidson K. W. (2020). Presenting characteristics, comorbidities, and outcomes among 5700 patients hospitalized with COVID-19 in the New York city area. JAMA 323 (20), 2052–2059. 10.1001/jama.2020.6775 32320003 PMC7177629

[B47] Shang J. Wan Y. Luo C. Ye G. Geng Q. Auerbach A. (2020). Cell entry mechanisms of SARS-CoV-2. Proc. Natl. Acad. Sci. U. S. A. 117 (21), 11727–11734. 10.1073/pnas.2003138117 32376634 PMC7260975

[B48] Shelton J. F. Shastri A. J. Ye C. Weldon C. H. Filshtein-Sonmez T. Coker D. (2021). Trans-ancestry analysis reveals genetic and nongenetic associations with COVID-19 susceptibility and severity. Nat. Genet. 53 (6), 801–808. 10.1038/s41588-021-00854-7 33888907

[B49] Shi G. Kenney A. D. Kudryashova E. Zani A. Zhang L. Lai K. K. (2021). Opposing activities of IFITM proteins in SARS-CoV-2 infection. EMBO J. 40 (3), e106501. 10.15252/embj.2020106501 33270927 PMC7744865

[B50] Wang F. Huang S. Gao R. Zhou Y. Lai C. Li Z. (2020). Initial whole-genome sequencing and analysis of the host genetic contribution to COVID-19 severity and susceptibility. Cell Discov. 6 (1), 83. 10.1038/s41421-020-00231-4 33298875 PMC7653987

[B51] Wang S. Wei M. Han Y. Zhang K. He L. Yang Z. (2008). Roles of TNF-alpha gene polymorphisms in the occurrence and progress of SARS-cov infection: A case-control study. BMC Infect. Dis. 8, 27. 10.1186/1471-2334-8-27 18312678 PMC2291466

[B52] Waters J. P. Pober J. S. Bradley J. R. (2013). Tumour necrosis factor in infectious disease. J. Pathol. 230 (2), 132–147. 10.1002/path.4187 23460469

[B53] Wiehagen K. R. Corbo-Rodgers E. Li S. Staub E. S. Hunter C. A. Morrisey E. E. (2012). Foxp4 is dispensable for T cell development, but required for robust recall responses. PloS one 7 (8), e42273. 10.1371/journal.pone.0042273 22912696 PMC3418275

[B54] Wu P. Ding L. Li X. Liu S. Cheng F. He Q. (2021). Trans-ethnic genome-wide association study of severe COVID-19. Commun. Biol. 4 (1), 1034. 10.1038/s42003-021-02549-5 34465887 PMC8408224

[B55] Wu Z. McGoogan J. M. (2020). Characteristics of and important lessons from the coronavirus disease 2019 (COVID-19) outbreak in China: Summary of a report of 72 314 cases from the Chinese center for disease control and prevention. JAMA 323 (13), 1239–1242. 10.1001/jama.2020.2648 32091533

[B56] Zeberg H. Pääbo S. (2020). The major genetic risk factor for severe COVID-19 is inherited from Neanderthals. Nature 587 (7835), 610–612. 10.1038/s41586-020-2818-3 32998156

[B57] Zhang H. Shao L. Lin Z. Long Q-X. Yuan H. Cai L. (2022). APOE interacts with ACE2 inhibiting SARS-CoV-2 cellular entry and inflammation in COVID-19 patients. Signal Transduct. Target. Ther. 7 (1), 261. 10.1038/s41392-022-01118-4 35915083 PMC9340718

[B58] Zhang Q. Bastard P. Liu Z. Le Pen J. Moncada-Velez M. Chen J. (2020)., 370. New York, NY), eabd4570. 10.1126/science.abd4570 Inborn errors of type I IFN immunity in patients with life-threatening COVID-19 Science 6515 32972995 PMC7857407

[B59] Zhang Y. Qin L. Zhao Y. Zhang P. Xu B. Li K. (2020). Interferon-induced transmembrane protein 3 genetic variant rs12252-C associated with disease severity in coronavirus disease 2019. J. Infect. Dis. 222 (1), 34–37. 10.1093/infdis/jiaa224 32348495 PMC7197559

[B60] Zhou F. Yu T. Du R. Fan G. Liu Y. Liu Z. (2020). Clinical course and risk factors for mortality of adult inpatients with COVID-19 in wuhan, China: A retrospective cohort study. Lancet 395 (10229), 1054–1062. 10.1016/S0140-6736(20)30566-3 32171076 PMC7270627

[B61] Zhou P. Yang X-L. Wang X-G. Hu B. Zhang L. Zhang W. (2020). A pneumonia outbreak associated with a new coronavirus of probable bat origin. Nature 579 (7798), 270–273. 10.1038/s41586-020-2012-7 32015507 PMC7095418

[B62] Zhu N. Zhang D. Wang W. Li X. Yang B. Song J. (2020). A novel coronavirus from patients with pneumonia in China, 2019. N. Engl. J. Med. 382 (8), 727–733. 10.1056/NEJMoa2001017 31978945 PMC7092803

[B63] Zlotnik A. Yoshie O. (2012). The chemokine superfamily revisited. Immunity 36 (5), 705–716. 10.1016/j.immuni.2012.05.008 22633458 PMC3396424

