# Supplementary figures and images for: Targeted screening of genetic associations with COVID-19 susceptibility and severity
Source: Front Genet. 2022 Nov 30;13:1073880. doi: 10.3389/fgene.2022.1073880 (PMC9747945; doi:10.3389/fgene.2022.1073880)

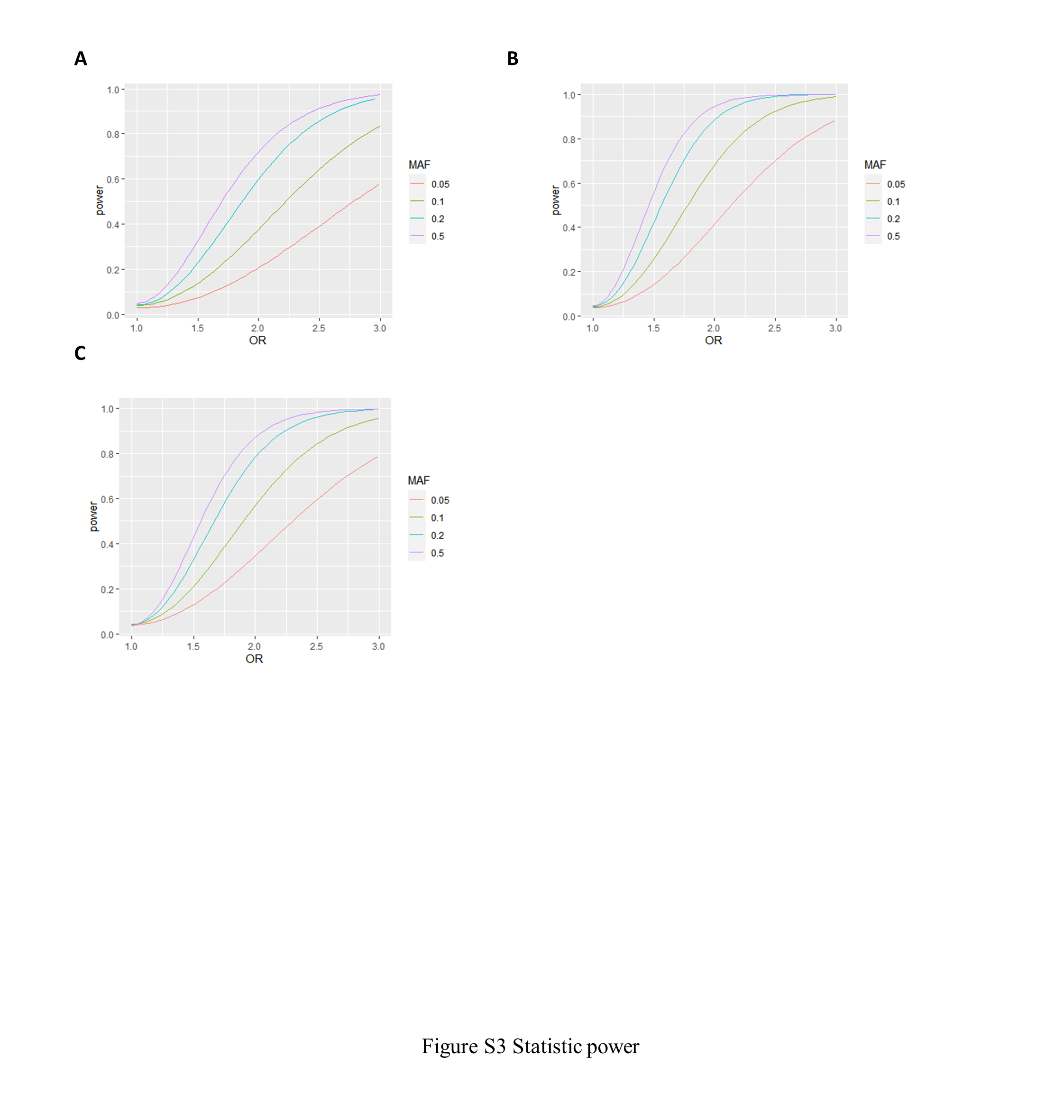

Supplement: Supplementary file 1 [file Image3.TIF]

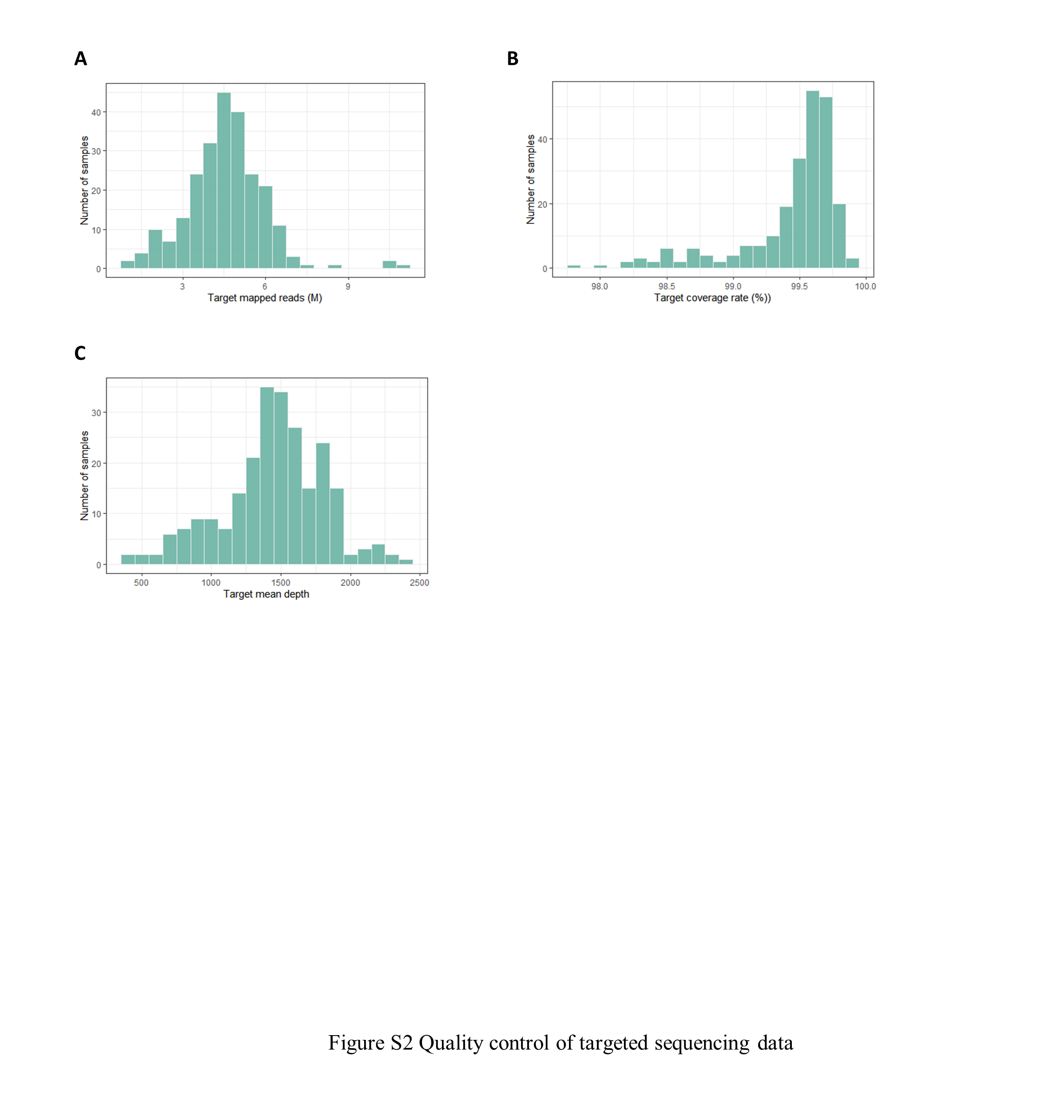

Supplement: Supplementary file 2 [file Image2.TIF]

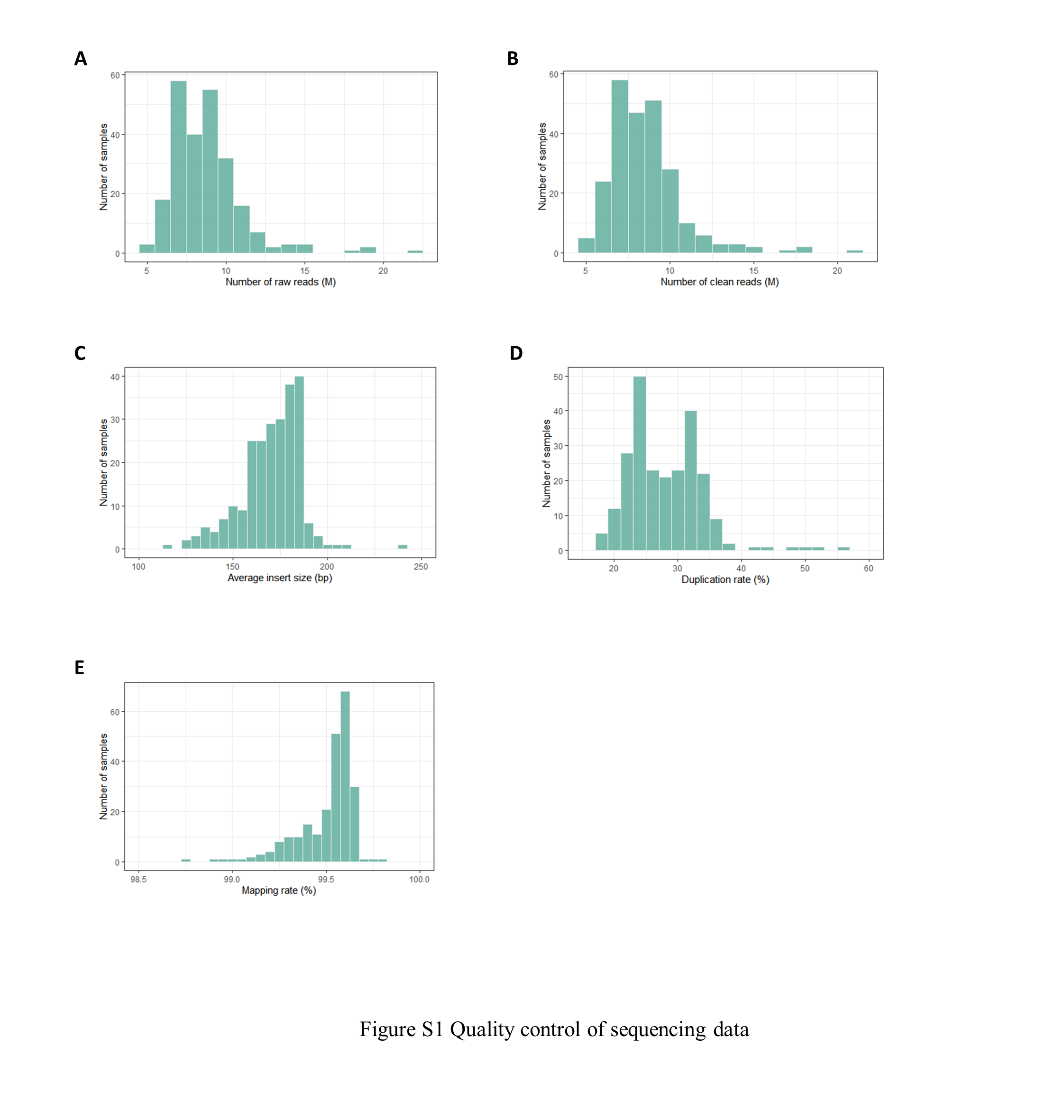

Supplement: Supplementary file 3 [file Image1.TIF]
